# Supplementary material for: Rapid categorization of natural face images in the infant right hemisphere
Source: eLife. 2015 Jun 2;4:e06564. doi: 10.7554/eLife.06564 (PMC4450157; doi:10.7554/eLife.06564)
Supplement: Supplementary file 1. — Table 1A. All 32 electrodes from Experiment 1, ranked by significance (p < 0.05, Bonferroni corrected for the number of channels: p < 0.00156, Z > 2.94). Significant responses were recorded at 5 channels on grand averaged data. Table 1B. All 32 electrodes from Experiment 2 ranked by significance. A significant response (p < 0.01, uncorrected) was recorded at channel P8, only for natural images. DOI: http://dx.doi.org/10.7554/eLife.06564.011 [file elife06564s001.docx]

**Supplemental File 1A**

| Electrode label | SNR | Z-score |
| --- | --- | --- |
| P8 | 2.56 | **12.16** |
| O1 | 1.14 | **4.17** |
| F3 | 1.16 | **3.63** |
| F7 | 1.24 | **3.62** |
| P7 | 1.47 | **3.61** |
| Cz | 1.57 | 2.94 |
| CP1 | 1.52 | 2.78 |
| FC1 | 1.08 | 2.78 |
| CP6 | 0.99 | 2.01 |
| F4 | 1.02 | 1.90 |
| FC2 | 1.38 | 1.67 |
| CP2 | 1.33 | 1.27 |
| FC5 | 1.13 | 1.17 |
| Fz | 1.26 | 1.03 |
| C4 | 1.27 | 0.87 |
| O2 | 1.38 | 0.87 |
| PO3 | 1.25 | 0.85 |
| CP5 | 0.97 | 0.24 |
| Fp1 | 1.03 | -0.20 |
| T7 | 0.92 | -0.24 |
| AF4 | 1.20 | -0.27 |
| PO4 | 1.13 | -0.32 |
| Oz | 1.05 | -0.44 |
| FC6 | 0.79 | -0.45 |
| P3 | 1.11 | -0.45 |
| Pz | 1.20 | -0.58 |
| AF3 | 0.99 | -0.69 |
| C3 | 1.21 | -0.78 |
| T8 | 1.20 | -0.90 |
| Fp2 | 1.22 | -0.94 |
| F8 | 1.10 | -1.31 |
| P4 | 0.88 | -1.31 |

**Table 1A**. All 32 electrodes from Experiment 1, ranked by significance (p < .05, Bonferroni corrected for the number of channels: p < .00156, Z > 2.94). Significant responses were recorded at 5 channels on grandaveraged data.

**Supplemental File 1B**

|  | **NATURAL IMAGES** | | | **SCRAMBLED IMAGES** | | |
| --- | --- | --- | --- | --- | --- | --- |
| **Electrode** | **SNR** | | **Z-score** | **SNR** | **Z-score** | |
| P8 | **2.09** | 2.01 | | 0.78 | | -0.81 |
| PO4 | 1.31 | 1.00 | | 0.90 | | -0.08 |
| C4 | 1.23 | 0.47 | | 1.19 | | -0.11 |
| AF3 | 1.14 | 0.44 | | 1.06 | | -0.02 |
| O1 | 1.40 | 0.33 | | 0.99 | | 0.45 |
| P7 | 1.09 | 0.29 | | 0.77 | | -0.88 |
| O2 | 1.09 | 0.18 | | 0.97 | | -0.39 |
| FC2 | 1.12 | 0.09 | | 0.91 | | -0.15 |
| Fp1 | 1.05 | 0.04 | | 1.06 | | 0.07 |
| Fz | 1.13 | 0.01 | | 1.10 | | 0.23 |
| FC1 | 1.02 | -0.02 | | 0.87 | | -0.57 |
| Oz | 1.22 | -0.03 | | 1.16 | | 0.49 |
| Fp2 | 1.02 | -0.04 | | 1.27 | | 0.54 |
| Pz | 0.99 | -0.07 | | 0.97 | | -0.67 |
| Cz | 1.03 | -0.08 | | 0.97 | | -0.35 |
| PO3 | 0.98 | -0.10 | | 1.01 | | 0.62 |
| F7 | 0.90 | -0.12 | | 1.13 | | 0.11 |
| CP2 | 0.91 | -0.29 | | 1.20 | | 0.46 |
| F3 | 0.90 | -0.29 | | 1.06 | | 0.06 |
| CP5 | 0.80 | -0.35 | | 1.19 | | 0.54 |
| FC5 | 0.83 | -0.35 | | 1.18 | | 0.38 |
| F4 | 0.87 | -0.37 | | 1.05 | | -0.09 |
| CP1 | 0.86 | -0.52 | | 0.76 | | -1.05 |
| FC6 | 0.86 | -0.54 | | 1.13 | | 0.34 |
| T7 | 0.84 | -0.56 | | 41.28 | | -0.38 |
| T8 | 0.93 | -0.62 | | 0.99 | | -0.02 |
| CP6 | 0.86 | -0.63 | | 0.94 | | -0.32 |
| AF4 | 0.79 | -0.64 | | 1.14 | | 0.25 |
| F8 | 0.66 | -0.68 | | 1.21 | | 0.68 |
| C3 | 0.80 | -0.77 | | 1.05 | | 0.28 |
| P3 | 0.71 | -0.84 | | 1.23 | | 0.32 |
| P4 | 0.98 | -0.84 | | 0.78 | | -0.64 |

**Table 1B**. All 32 electrodes from Experiment 2 ranked by significance. A significant response (p<0.01, uncorrected) was recorded at channel P8, only for natural images.
